# ﻿A comparative study of the external male genitalia in the subfamily Zeugophorinae, and a new species of genus *Zeugophora* (Coleoptera, Megalopodidae) from China

**DOI:** 10.3897/zookeys.1261.169189

**Published:** 2025-12-02

**Authors:** Kai-Qin Li, Yuan Xu, Hong-Bin Liang

**Affiliations:** 1 Kunming Natural History Museum of Zoology, Kunming Institute of Zoology, Chinese Academy of Sciences, Kunming 650223, China; 2 Yunnan Key Laboratory of Biodiversity Information, Kunming Institute of Zoology, Chinese Academy of Sciences, Kunming 650201, China; 3 College of Forestry, Southwest Forestry University, Kunming 650224, China; 4 Key Laboratory of Zoological Systematics and Evolution, Institute of Zoology, Chinese Academy of Sciences, Beijing 100101, China

**Keywords:** Key, median lobe, median struts, *
Pedrillia*, spiculum, tegmen

## Abstract

The external male genitalia of 21 Chinese species of the genus *Zeugophora* (subfamily Zeugophorinae) are compared. A key is compiled to Chinese *Zeugophora* species based on external male genitalia with aid of other external morphological traits. One new species, Zeugophora (Pedrillia) liupanshanensis Li & Liang, **sp. nov.**, is described from Ningxia Hui Autonomous Region, China.

## ﻿Introduction

The external male genitalia of Megalopodidae consist of a median lobe, a pair of median struts, a tegmen, an internal sac and a spiculum (belonging to the 9^th^ abdominal segment) (Snodgrass 1935; Chen 1940; Verma 1996). They are among the most important identification characteristics in the Megalopodidae and receive much attention from taxonomists. Chûjô (1952, 1953) studied the morphology of external male genitalia of the Megalopodinae and Zeugophorinae extensively and found similarities between these two subfamilies. Subsequently, taxonomists began to pay attention to the male genitalia of the Megalopodidae when describing new species and constructing phylogenetic relationships within the Chrysomeloidea (Reid 1989, 1998; Kuschel and May 1990; Mann and Crowson 1996; Verma 1996; Medvedev 1997; Li et al. 2013; Li and Liang 2018, 2020; Rodríguez-Mirón et al. 2021).

The current classification of the genus *Zeugophora* is unstable. Some authors treated *Pedrillia* Westwood, 1864 as a distinct genus of the Zeugophorinae (Baly 1873; Jacoby 1908; Chûjô 1935, 1937) or as a subgenus of *Zeugophora* (Monrós 1959; Gressitt and Kimoto 1961; Chen and Pu 1962; Kimoto and Gressitt 1979; Medvedev 1985, 1997; Reid 1989, 1992, 1995; Schöller 2009; Silfverberg 2010; Warchałowshi 2010; Li and Liang 2018, 2020), while other authors treated *Pedrillia* as a synonym of the genus *Zeugophora* (Sekerka and Vives 2013; Rodríguez-Mirón, 2018; Takemoto, 2019; Rodríguez-Mirón et al. 2021). We accept that Pedrillia is a subgenus of the genus Zeugophora because *Pedrillia* can be separated from *Zeugophora* on external morphology, external male and female genitalia, and host plants, at least for Chinese species. A total of 34 species of subfamily Zeugophorinae has been recorded in China (Lee and Cheng 2007; Li and Liang 2018, 2020). In *Pedrillia*, it is difficult to determine certain species based only on external morphological characteristics; therefore, we attempted to find characteristics of external male genitalia to aid its classification. The comparison of female genitalia was not included in this study owing to insufficient specimen material in several species. In this study, we describe and compare 21 available Chinese species of Zeugophorinae to summarize key characteristics of external male genitalia for distinguishing subgenera and species within the Chinese fauna.

With accumulating information about the host plants of Zeugophorinae during expeditions in China, we found that plants of the family Celastraceae are hosted by a few species, such as Zeugophora (Pedrillia) annulata (Baly, 1873), Z. (P.) bicolor (Kraatz, 1879), Z. (P.) nigricollis (Jacoby, 1885), Z. (P.) ruficollis Chûjô, 1932, Z. (P.) decorata Chûjô, 1937, Z. (P.) formosana (Gressitt, 1945), Z. (P.) tricolor Chen & Pu, 1962, Z. (P.) euonymorum Li & Liang, 2020 (Lee and Cheng 2007; Li and Liang 2018, 2020). In recent years, we focused on trees of Celastraceae when surveying Zeugophorinae. In 2018, two specimens were collected by beating plants of the family Celastraceae in Ningxia Hui Autonomous Region. We compared them with all known species, and found they represented a new species, described in this paper.

## ﻿Materials and methods

### ﻿Preparation of specimens

Dry specimens were soaked in water for 1–2 h. The lateral margin of the abdomen was opened and the genitalia were pulled out of the abdomen with fine forceps. The genitalia were soaked in a warm solution of 10% KOH for 10–20 min. After treatment, these organs were washed with water. The genitalia were then detached and transferred to glycerin for observation, photography, and preservation.

All measurements were made using a Nikon SMZ1500 or Nikon SMZ18 stereoscopic microscope with the aid of an ocular micrometer. Body length (**BL**) = the linear distance along the midline from the anterior margin of the labrum to the apex of the elytra; body width (**BW**) = elytra width (**EW**) = the maximum linear distance across the elytra; pronotum length (**PL**) = the linear distance along the median line of the pronotum; pronotum width (**PW**) = the linear distance across the widest part of the pronotum; elytra length (**EL**) = the linear distance from the base of the elytra to the apex of the sutural angle; median lobe length = the linear distance from the base to the apex; median struts length = the linear distance from the base to the apex. Ratios cited in descriptions are based on these measurements.

Photographs of male genitalia were taken using a Nikon SMZ-1500 stereoscopic dissecting microscope fitted with a Cannon 450D digital camera, or a Nikon SMZ18 stereoscopic dissecting microscope fitted with a Nikon D610 digital camera. For each final image, several photographs were taken using different focal planes, combined with Helicon Focus software to obtain one synthesized photograph, and finally edited with Adobe Photoshop software.

### ﻿Terminology

Morphological terminology for male genitalia follows Snodgrass (1935), Verma (1996), Chûjô (1952, 1953), Kasap and Crowson (1985), Li and Liang (2018, 2020), and Li et al. (2013).

### ﻿Abbreviations and acronyms

The abbreviation **TL** means type locality. The abbreviation **TD** means type depository.

The abbreviations used in the descriptions are as follows:

**BFU**Beijing Forestry University, Beijing, China.

**CAS**California Academy of Sciences, San Francisco, California, USA.

**IZCAS**National Zoological Museum of China, Institute of Zoology, Chinese Academy of Sciences, Beijing, China.

**KIZ**Kunming Natural History Museum of Zoology, Kunming Institute of Zoology, Chinese Academy of Sciences, Kunming, China.

**MZPW**Polish Academy of Science, Museum of the Institute of Zoology, Warsaw, Poland.

**NHMUK**The Natural History Museum, London, UK.

**NSMK**National Science Museum, Korea.

**SCBG**Shanghai Chenshan Botanical Garden, Shanghai, China.

**SDEI**Deutsches Entomologisches Institut, Eberswalde, Germany.

**ZIN**Zoological Institute, Russian Academy of Sciences, St. Petersburg, Russia.

### ﻿Species studied

A total of 21 available *Zeugophora* (subgenera *Zeugophora* and *Pedrillia*) species belonging subfamily Zeugophorinae were examined (Table 1).

**Table 1.** Species list of Zeugophorinae studied in this paper.

Species	Locality	Specimen status	Host plant
Zeugophora (Zeugophora) ancora Reitter, 1900	Ningxia	common	Salicaceae: Populus nigra var. italica (Moench) Koehne, *Populus alba* L.
Zeugophora (Z.) cribrata Chen, 1974	Qinghai	paratype	Salicaceae: *Salix* L.
Zeugophora (Z.) cyanea Chen, 1974	Sichuan, Qinghai	specimen compared with holotype	Salicaceae: *Salix* L.
Zeugophora (Z.) nigroaerea Lopatin, 2008	Gansu, Shaanxi	specimen compared with holotype	Salicaceae: *Salix* L.
Zeugophora (Z.) scutellaris Suffrian, 1840	Inner Mongolia	common	Salicaceae: *Populus acuminata* Rydberg, *P. alba* L., *P. deltoides* Marshall, *P. grandidentata* Michaux, *P. nigra* L., *P. tremula* L., *Salix* sp.
Zeugophora (Z.) turneri Power, 1863	Beijing	common	Salicaceae: *Populus* sp.
Zeugophora (Pedrillia) annulata (Baly, 1873)	Ningxia	common	Celastraceae: *Euonymus alatus* (Thunb.) Siebold, *Euonymus przewalskii* Maxim., *Euonymus phellomanus* Loes. ex Diels. *Euonymus maacki* Rupr. and *E. sacrosancta* Koidz.
Zeugophora (P.) bicolor (Kraatz, 1879)	Liaoning	specimen compared with holotype	Celastraceae: *Celastrus orbiculatus* Thunb.
Zeugophora (P.) dimorpha Gressitt, 1945	Hunan	specimen compared with paratype	unknown
Zeugophora (P.) emeica Li & Liang, 2018	Sichuan	holotype	unknown
Zeugophora (P.) euonymorum Li & Liang, 2020	Yunnan	holotype	Celastraceae: *Euonymus alatus* (Thunb.) Siebold, *Euonymus hamiltonianus* Wall.
Zeugophora (P.) flavithorax Li & Liang, 2020	Yunnan	holotype	Symplocaceae
Zeugophora (P.) indica Jacoby, 1903	Yunnan	specimen compared with holotype	possible Lamiaceae: *Vitex quinata* (Lour.) Will.
Zeugophora (P.) longicornis (Westwood, 1864)	Yunnan	specimen compared with type	possible Lamiaceae: *Vitex quinata* (Lour.) Will.
Zeugophora (P.) maculata Chûjô, 1941	Guizhou	common	Symplocaceae: *Symplocos sumuntia* Buch.-Ham. ex D. Don
Zeugophora (P.) nigricollis (Jacoby, 1885)	Ningxia	common	Celastraceae: *Euonymus alatus* (Thunb.) Siebold
Zeugophora (P.) nigroapica Li & Liang, 2018	Yunnan	holotype	unknown
Zeugophora (P.) tricolor Chen & Pu, 1962	Ningxia	specimen compared with holotype	Celastraceae: *Euonymus alatus* (Thunb.) Siebold
Zeugophora (P.) trifasciata Li & Liang, 2020	Yunnan	holotype	unknown
Zeugophora (P.) yuae Li & Liang, 2020	Yunnan	holotype	possible Lamiaceae: *Vitex quinata* (Lour.) Will.
Zeugophora (P.) yunnanica Chen & Pu, 1962	Yunnan	specimen compared with holotype	Symplocaceae: *Symplocos* sp.

## ﻿Results

### ﻿Key to species of the genus *Zeugophora*

**Table:** 

1	Median strut short, ratio of median strut / median lobe ≤ 1.5 (subgenus Zeugophora)	**2**
–	Median strut long, ratio of median strut / median lobe ≥ 2.0 (subgenus Pedrillia)	**7**
2	Median lobe with apical portion gradually narrowed towards apex, apex blunt or sharp (Figs 2, 18, 23); host plant genus *Populus*	**3**
–	Median lobe with apical portion abruptly narrowed towards apex, apex sharp (Figs 7, 10, 15); host plant genus *Salix*	**5**
3	Median lobe with apical portion broad, apex blunt (Fig. 2)	**Zeugophora (Z.) ancora Reitter**
–	Median lobe with apical portion narrow, apex sharp (Figs 18, 23)	**4**
4	Dorso-central portion of median lobe without a sclerite (Fig. 18)	**Zeugophora (Z.) scutellaris Suffrian**
–	Dorso-central portion of median lobe with a sclerite (Fig. 23)	**Zeugophora (Z.) turneri Power**
5	Apical portion of median lobe slightly broad (Fig. 7)	**Zeugophora (Z.) cribrata Chen**
–	Apical portion of median lobe slightly narrow (Figs 10, 15)	**6**
6	Apex of median lobe slightly long (Fig. 15)	**Zeugophora (Z.) nigroarea Lopatin**
–	Apex of median lobe slightly short (Fig. 10)	**Zeugophora (Z.) cyanea Chen**
7	Sides of the median lobe narrow in basal and apical portion (Figs 39, 65)	**8**
–	Sides of median lobe parallel or sub-parallel	**9**
8	Median basal portion lobe broader, apex strongly narrowed and bent downward;median strut short; tegmen strongly sclerotized (Figs 38–40)	**Zeugophora (P.) emeica Li & Liang**
–	Median lobe elongate, apex slightly swollen laterally; median strut long; tegmen slightly sclerotized (Figs 64–66)	**Zeugophora (P.) nigroapica Li & Liang**
9	Sides of median lobe parallel; dorso-central portion of median lobe with a sclerite (Figs 34, 73)	**10**
–	Sides of median lobe parallel, sub-parallel or not parallel; dorso-central portion of median lobe without a sclerite	**11**
10	Sclerite of median lobe in dorso-central area weak; sides of spiculum longer than central portion (Figs 34, 36)	**Zeugophora (P.) dimorpha Gressitt**
–	Sclerite of median lobe in dorso-central area strong; sides of spiculum shorter than central portion (Figs 72, 75)	**Zeugophora (P.) trifasciata Li & Liang**
11	Median lobe with dorso-central portion membranous, basal 1/3 not tubular; the sides of median lobe thin (Figs 47, 58, 82); host plant Symplocaceae	**12**
–	Median lobe with dorso-central portion membranous, basal 1/3 tubular; sides of median lobe thick; host plant Lamiaceae or Celastraceae	**14**
12	Ratio of median strut / median lobe ~3.0; apex of median lobe sharp (Figs 81, 82); apical portion of spiculum trifid	**Zeugophora (P.) yunnanica Chen & Pu**
–	Ratio of median strut / median lobe 1.5–2.0; apex of median lobe blunt or prominent; apical portion of spiculum not trifid (Figs 46, 47, 57, 58)	**13**
13	Median lobe apical portion slightly broad, apical margin projecting and flattened (Fig. 47)	**Zeugophora (P.) flavithorax Li & Liang**
–	Median lobe apical portion slightly narrow, apical margin blunt and bent downward medially (Fig. 58)	**Zeugophora (P.) maculata Chûjô**
14	Ratio of median strut / median lobe > 3.0; host plant Lamiaceae	**15**
–	Ratio of median strut / median lobe < 3.0; host plant Celastraceae	**17**
15	Median lobe strongly curved in lateral view, apical portion strongly upward curved, apex sharp (Figs 54, 55)	**Zeugophora (P.) longicornis (Westwood)**
–	Median lobe curved in lateral view, apical portion flattened, apex blunt	**16**
16	Median lobe elongate, sides slightly narrowed in middle, apex slightly short (Figs 76, 77)	**Zeugophora (P.) yuae Li & Liang**
–	Median lobe slightly broad and short, sides paralleled, apex slightly long (Figs 49, 50)	**Zeugophora (P.) indica Jacoby**
17	Median lobe basal portion curved in lateral view (Figs 41, 60, 92, 97)	**18**
–	Median lobe basal and apical portion curved in lateral view (Figs 25, 30, 69)	**20**
18	Media lobe short, apex broad (Figs 41, 42)	**Zeugophora (P.) euonymorum Li & Liang**
–	Median lobe elongate, apex narrow	**19**
19	Apical portion of median lobe broad (Fig. 61)	**Zeugophora (P.) nigricollis (Jacoby)**
–	Apical portion of median lobe narrow (Figs 93, 98)	**Zeugophora (P.) liupanshanensis sp. nov.**
20	Apex of median lobe slightly curved Leftward (Fig. 31), basal piece of tegmen long (Fig. 32)	**Zeugophora (P.) bicolor (Kraatz)**
–	Apex of median lobe not curved Leftward (Figs 26, 70), basal piece of tegmen short (Figs 27, 71)	**21**
21	Apex of the median lobe long and sharp (Fig. 26)	**Zeugophora (P.) annulata (Baly)**
–	Apex of median lobe short and blunt (Fig. 70)	**Zeugophora (P.) tricolor Chen & Pu**

### ﻿Zeugophorinae Boving & Craighead, 1931

**Genus*Zeugophora* Kunze, 1818**

#### ﻿ Subgenus Zeugophora

Taxon classificationAnimaliaColeopteraMegalopodidae

Kunze, 1818

EC31E345-4B73-5655-AD7D-0AACFDC84697

##### External male genitalia.

(Figs 1–24) Median lobe generally strongly sclerotized, slender, dorso-central portion membranous with or without a long narrow sclerite, curved in lateral view, sides thin, sub-parallel, apex narrower than base, apical portion tongue shaped, blunt, or triangular and sharp; median struts rod-shaped, widely separated from each other, ~1.1–1.4× as long as median lobe; base of tegmen slightly Y-shaped, tegminal ring with base broad and gradually narrow to apical portion, apex sub-square, paramere sub-square, apical margin with dense setae; endophallus membranous, with a paired granulated area, sclerotized or weakly sclerotized. Spiculum long, Y-shaped, strongly sclerotized, apical portion trifid, slightly sclerotized.

The apical portion of median lobe with a long narrow sclerite in Zeugophora (Zeugophora) ancora, Z. (Z.) cribrata, Z. (Z.) cyanea, Z. (Z.) nigroaerea, and Z. (Z.) turneri (Figs 2, 7, 10, 15, 23) but not in Z. (Z.) scutellaris (Fig. 18).

#### ﻿ Zeugophora (Zeugophora) ancora

Taxon classificationAnimaliaColeopteraMegalopodidae

Reitter, 1900

D5B6B0D8-35C0-5A78-A7F6-2491FD5BFBC3

Figs 1–4

Zeugophora ancora Reitter, 1900: 164, pl. 1, fig. 9. TL: C. Asia (Donkyr). TD: unknown.
Zeugophora ancora var. pseudancora Reitter, 1900: 165.
Zeugophora (Pedrillia) ancora : Jolivet 1957: 12.

##### Host plants.

Salicaceae: Populus nigra var. italica (Moench) Koehne, *Populus alba* L. (Wu et al. 1982).

##### Remarks.

The median lobe of this species is similar to that of Zeugophora (Zeugophora) cyanea, Z. (Z.). *cribrata*, Z. (Z.) nigroaerea, and Z. (Z.) turneri in having the dorso-central portion of the median lobe with a sclerite (Figs 7, 10, 15, 23), but differs in having the apex of median lobe blunt.

#### ﻿ Zeugophora (Zeugophora) cribrata

Taxon classificationAnimaliaColeopteraMegalopodidae

Chen, 1974

B33315AC-98E6-58D8-AEC6-96D07EA9C5E5

Figs 5–8

**Figures 1–8.**
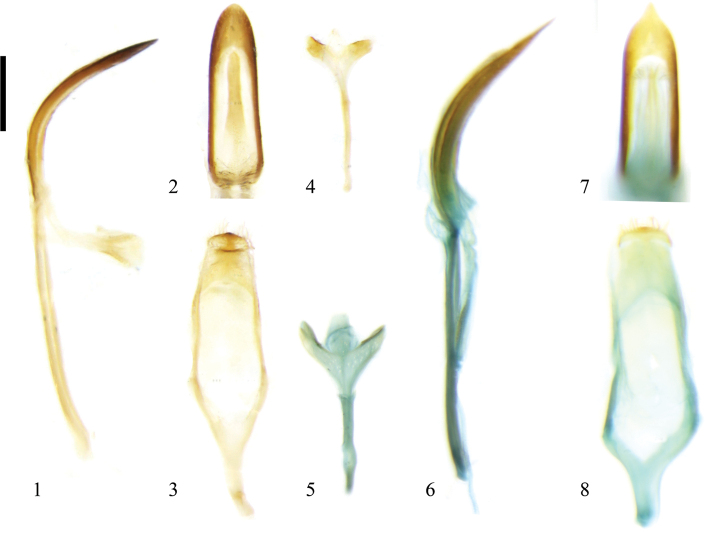
Male genitalia of Zeugophora (Zeugophora) species. **1–4.**Z. (Z.) ancora Reitter, 1900; **1.** Median lobe and median struts, lateral view; **2.** Median lobe, dorsal view; **3.** Tegmen, dorsal view; **4.** Spiculum, dorsal view; **5–8.**Z. (Z.) cribrata Chen, 1974; **5.** Spiculum, dorsal view; **6.** Median lobe and median struts, lateral view; **7.** Median lobe, dorsal view; **8.** Tegmen, dorsal view. Scale bar: 2 mm.

Zeugophora cribrata Chen, 1974: 43, 44, 47, fig. 1A. TL: China, Qinghai. TD: IZCAS.

##### Host plant.

Salicaceae: *Salix* L. (Chen 1974; pers. obs.).

##### Remarks.

The external male genitalia of this species are similar to those of Zeugophora (Zeugophora) cyanea, but differ in having the median lobe slightly broader (median lobe slightly narrower in Z. (Z.) cyanea, Fig. 10).

#### ﻿ Zeugophora (Zeugophora) cyanea

Taxon classificationAnimaliaColeopteraMegalopodidae

Chen, 1974

8FD6CF30-2264-5FF9-BA4D-A22B78B0BF6E

Figs 9–12

Zeugophora cyanea Chen, 1974: 44, 45, 47. TL: China, Qinghai. TD: IZCAS.

##### Host plant.

Salicaceae: *Salix* L. (Chen 1974; pers. obs.).

##### Remarks.

The similarities and differences of external male genitalia between this species and Zeugophora (Zeugophora) cribrata are provided in the remarks under Z. (Z.) cribrata.

#### ﻿ Zeugophora (Zeugophora) nigroaerea

Taxon classificationAnimaliaColeopteraMegalopodidae

Lopatin, 2008

F121FF25-4BDA-532A-86CB-60119C376879

Figs 13–16

Zeugophora nigroaerea Lopatin, 2008: 918–927. TL: China (Gansu). TD: ZIN.

##### Host plant.

Salicaceae: *Salix* L. (pers. obs.).

##### Remarks.

**Figures 9–16.**
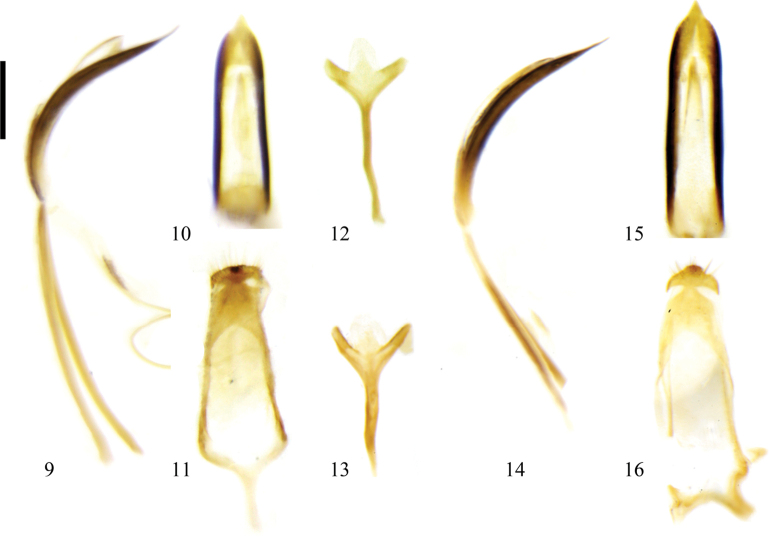
Male genitalia of Zeugophora (Zeugophora) species. **9–12.**Z. (Z.) cyanea Chen, 1974; **9.** Median lobe and median struts, lateral view; **10.** Median lobe, dorsal view; **11.** Tegmen, dorsal view; **12.** Spiculum, dorsal view; **13–16.**Z. (Z.) nigroaerea Lopatin, 2008; **13.** Spiculum, dorsal view; **14.** Median lobe and median struts, lateral view; **15.** Median lobe, dorsal view; **16.** Tegmen, dorsal view. Scale bar: 0.2 mm.

The external male genitalia of this species are most similar to those of Zeugophora (Zeugophora) cyanea (Figs 9–12), but differ in having the median lobe slightly longer (median lobe slightly shorter in Z. (Z.) cyanea, Fig. 10). This species is also similar to Z. (Z.) cribrata (Figs 5–8), but differs from it in having the apex of the median lobe slightly narrower (apex of median lobe slightly broader in Z. (Z.) cribrata, Fig. 7).

#### ﻿ Zeugophora (Zeugophora) scutellaris

Taxon classificationAnimaliaColeopteraMegalopodidae

Suffrian, 1840

FFB46474-DF79-5E4C-BFE1-6F2925B268DA

Figs 17–20

Zeugophora scutellaris Suffrian, 1840: 99. TL: Germany (Aschersleben, Magdeburg). TD: MZPW.

##### Host plants.

Salicaceae: *Populus acuminata* Rydberg, *P. alba* L., *P. deltoides* Marshall, *P. grandidentata* Michaux, *P. nigra* L., *P. tremula* L., *Salix* sp. (Medvedev and Rogynskaya 1988; Bukejs 2009; Sergeev and Legalov 2022).

##### Remarks.

The external male genitalia of this species differ from those of other species of the subgenus Zeugophora (Zeugophora) in having the dorso-central portion without a sclerite (Fig. 18).

#### ﻿ Zeugophora (Zeugophora) turneri

Taxon classificationAnimaliaColeopteraMegalopodidae

Power, 1863

A7E3E2C2-6A90-5080-8C9A-69464339E82D

Figs 21–24

Zeugophora turneri Power, 1863, 21: 8735. TL unknown. TD unknown.
Zeugophora rufotestacea Kraatz, 1871, 15: 162.

##### Host plant.

Salicaceae: *Populus* sp. (Bukejs 2009).

##### Remarks.

The external male genitalia of this species are similar to those of Zeugophora (Zeugophora) cribrata and Z. (Z.) cyanea in the dorso-central portion of median lobe with a sclerite, but differ in having apex of median lobe gradually narrowed (apex of median lobe abruptly narrowed in Z. (Z.) cribrata and Z. (Z.) cyanea).

#### ﻿ Subgenus Pedrillia

Taxon classificationAnimaliaColeopteraMegalopodidae

Westwood, 1864

EF2E7FB0-3884-5631-81DB-11AC553AE46B

##### External male genitalia.

(Figs 25–83, 92–99) Median lobe sclerotized, slender or short and broad, curved in lateral view, sides generally thickened, dorso-central portion entirely membranous, sides parallel or diamond-shaped, or dorso-central portion membranous with basal 1/3 tubular, sides parallel, apex generally strongly protruding, blunt or sharp; median struts rod-shaped, widely separated from each other, ~1.5–3.3× as long as median lobe; base of tegmen Y- or V-shaped, basal piece generally short, tegminal ring with base generally narrower than apical portion, paramere generally tongue-shaped or sub-trapezoid, apical margin with setae; endophallus membranous, with a paired granulated area. Spiculum long, basal part long, apex trifid or not, sclerotized.

#### ﻿ Zeugophora (Pedrillia) annulata

Taxon classificationAnimaliaColeopteraMegalopodidae

(Baly, 1873)

D2D6B911-9077-50A9-A414-62981636052F

Figs 25–28

Pedrillia annulata Baly, 1873: 79. TL: Japan. TD: NHMUK.
Pedrillia annulata var. disconotata Pic, 1906: 27. Synonymized by Ohno 1961: 37.
Pedrillia annulata ab. theresae Pic, 1945: 13.
Zeugophora annulata : Gressitt, 1945: 137.
Zeugophora (Pedrillia) annulata : Jolivet, 1957: 12.
Pedrillia annulata f. inannulata Chûjô, 1959: 1.
Pedrillia annulata f. melanaria Chûjô, 1959: 1.
Pedrillia biguttata Kraatz, 1879a: 119. Synonymized by Ohno, 1961: 37.
Pedrillia annulata f. biguttata : Ohno, 1961: 37.

##### Host plants.

Celastraceae: *Euonymus alatus* (Thunb.) Siebold, *Euonymus przewalskii* Maxim., *Euonymus phellomanus* Loes. ex Diels., *Euonymus maacki* Rupr., and *Euonymus sacrosancta* Koidz. (Medvedev and Roginskaya 1988; Takemoto 2019; pers. obs.).

##### Remarks.

The external male genitalia of this species are similar to those of Zeugophora (Pedrillia) bicolor and Z. (P.) tricolor in having the median lobe long and arched in lateral view, and the shapes of median lobe, tegmen, and spiculum. This species differs from Z. (P.) bicolor in having the apex of the median lobe slightly narrower, not bending / curving leftwards, basal piece of tegmen shorter (apex of median lobe slightly broader, tending slightly leftwards, basal piece of tegmen longer in Z. (P.) bicolor, Figs 31, 32). This species differs from Z. (P.) tricolor in having the apex of the median lobe slightly longer and sharper (apex of median lobe slightly shorter and blunt in Z. (P.) tricolor, Fig. 70).

**Figures 17–24.**
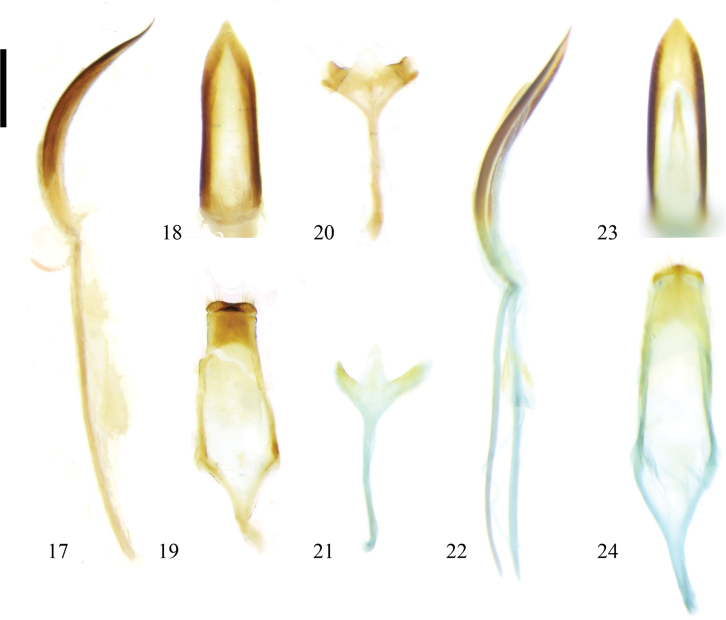
Male genitalia of Zeugophora (Zeugophora) species. **17–20.**Z. (Z.) scutellaris Suffrian, 1840; **17.** Median lobe and median struts, lateral view; **18.** Median lobe, dorsal view; **19.** Tegmen, dorsal view; **20.** Spiculum, dorsal view; **21–24.**Z. (Z.) turneri Power, 1863; **21.** Spiculum, dorsal view; **22.** Median lobe and median struts, lateral view; **23.** Median lobe, dorsal view; **24.** Tegmen, dorsal view. Scale bar: 0.2 mm.

#### ﻿ Zeugophora (Pedrillia) bicolor

Taxon classificationAnimaliaColeopteraMegalopodidae

(Kraatz, 1879)

417E7363-56FF-53DC-92B0-C0815DF9668F

Figs 29–32

Pedrillia bicolor Kraatz, 1879a: 120. TL: Russia (Amur). TD: SDEI.
Pedrillia nigricollis Jacoby, 1885: 195. Synonymized by Kimoto 1986: 309.
Zeugophora bicolor : Gressitt 1945: 139.
Zeugophora (Pedrillia) bicolor : Jolivet 1957: 12.

##### Host plant.

Celastraceae: *Celastrus orbiculatus* Thunb. (pers. obs.).

##### Remarks.

The similarities and differences of external male genitalia between this species and Zeugophora (Pedrillia) annulata are provided in the remarks under Z. (P.) annulata.

The external male genitalia of this species are also similar to Zeugophora (Pedrillia) in having the median lobe long and arched in lateral view, and the shapes of the median lobe, tegmen, and spiculum, but differs from it in having the apex of the median lobe longer and narrower (apex of median lobe shorter and broader in Z. (P.) tricolor, Fig. 70).

**Figures 25–32.**
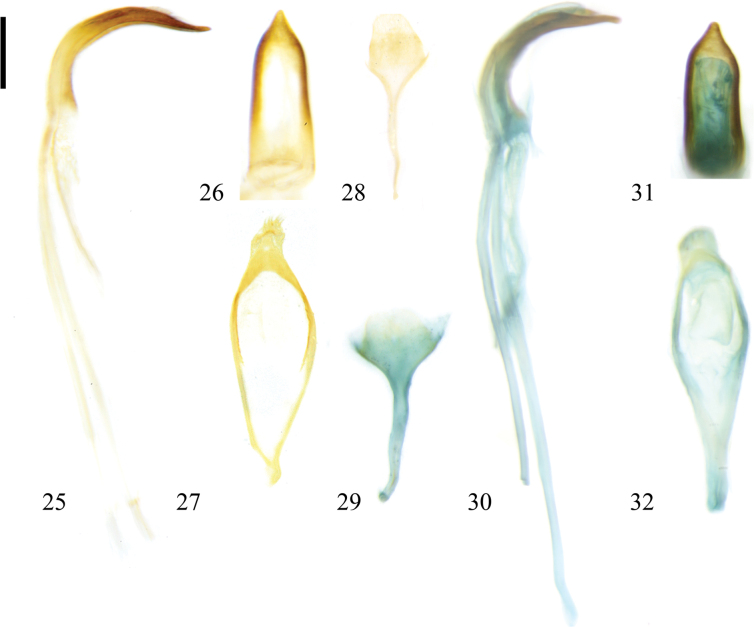
Male genitalia of Zeugophora (Pedrillia) species. **25–28.**Z. (P.) annulata (Baly, 1873); **25.** Median lobe and median struts, lateral view; **26.** Median lobe, dorsal view; **27.** Tegmen, dorsal view; **28.** Spiculum, dorsal view; **29–32.**Z. (P.) bicolor (Kraatz, 1879); **29.** Spiculum, dorsal view; **30.** Median lobe and median struts, lateral view; **31.** Median lobe, dorsal view; **32.** Tegmen, dorsal view. Scale bar: 0.2 mm.

#### ﻿ Zeugophora (Pedrillia) dimorpha

Taxon classificationAnimaliaColeopteraMegalopodidae

Gressitt, 1945

4B8D1000-53BE-5A0D-ADDF-ACEC3EF290EE

Figs 33–36

Zeugophora dimorpha Gressitt, 1945: 138, pl. 6, figs 6, 9. TL: China: (Jiangxi, Guangdong). TD: CAS.
Zeugophora (Pedrillia) dimorpha : Jolivet 1957: 12.

##### Host plant.

Unknown.

##### Remarks.

The external male genitalia of this species are similar to those of Zeugophora (Pedrillia) yunnanica Chen & Pu and Z. (P.) trifasciata Li & Liang in shape, but differ from Z. (P.) yunnanica in having the median lobe more slender, the sides of the spiculum longer than the central portion (median lobe shorter and broader, the central portion of spiculum longer than sides in Z. (P.) yunnanica, Figs 80, 82), differ from Z. (P.) trifasciata in having the sclerite of median lobe dorso-central slightly sclerotized and the sides of the spiculum longer than the central portion (strongly sclerotized and the central portion of spiculum longer than sides in Z. (P.) trifasciata).

#### ﻿ Zeugophora (Pedrillia) emeica

Taxon classificationAnimaliaColeopteraMegalopodidae

Li & Liang, 2018

1A5BBE42-80D5-5672-BEA6-921887387F83

Figs 37–40

Zeugophora (Pedrillia) emeica Li & Liang, 2018: 135. TL: China (Sichuan). TD: IZCAS.

##### Host plant.

Unknown.

##### Remarks.

The external male genitalia of this species are similar to that of Zeugophora (Pedrillia) nigroapica in having the base and apex of the median lobe narrow, but differ in having the base of the median lobe broader, apex strongly narrowed and downward, median strut short, tegmen strongly sclerotized (median lobe elongate, apex slightly swollen laterally, median struts long, tegmen slightly sclerotized in Z. (P.) nigroapica, Figs 64–66).

#### ﻿ Zeugophora (Pedrillia) euonymorum

Taxon classificationAnimaliaColeopteraMegalopodidae

Li & Liang, 2020

97CFF1AB-EBAA-5A73-A1ED-D699C339F578

Figs 41–44

Zeugophora (Pedrillia) euonymorum Li & Liang, 2020, ZooKeys, 975: 57–61, figs 12–28. TL China: Ningxia. TD: KIZ, IZCAS.

##### Host plants.

Celastraceae: *Euonymus alatus* (Thunb.) Siebold, *E. hamiltonianus* Wall. (Li and Liang 2020).

##### Remarks.

The external male genitalia of this species are similar to those of Zeugophora (Pedrillia) annulata, Z. (P.) bicolor, Z. (P.) tricolor, but differ from them in having a slightly flattened and less curved median lobe in the lateral view, the apex of the median lobe shorter and slightly broader.

#### ﻿ Zeugophora (Pedrillia) flavithorax

Taxon classificationAnimaliaColeopteraMegalopodidae

Li & Liang, 2020

A35D9DB4-B651-51BE-9A13-67709ACA2163

Figs 45–48

Zeugophora (Pedrillia) flavithorax Li & Liang, 2020, ZooKeys, 975: 61–66, figs 29–37, 46–47. TL China: Guizhou. TD: KIZ, IZCAS.

##### Host plant.

Symplocaceae (Li and Liang 2020).

##### Remarks.

The external male genitalia of this species differ from those of other species of Zeugophora (Pedrillia) in having the median lobe apex slightly broad and the apical margin projecting.

#### ﻿ Zeugophora (Pedrillia) indica

Taxon classificationAnimaliaColeopteraMegalopodidae

Jacoby, 1903

83653916-E7D8-52ED-A322-8534D0637027

Figs 49–52

Zeugophora indica Jacoby, 1903: 81–82. TL: India (Nilgiri). TD: NHMUK.
Pedrillia flavipes Jacoby, 1908: 14. Synonymized by Bryant 1943: 246.
Auehenia (Pedrillia) indica : Monrós 1959: 23.
Zeugophora (Pedrillia) indica : Kimoto & Gressitt 1979: 205.

##### Host plant.

Possibly Lamiaceae: *Vitex quinata* (Lour.) Will. (pers. obs.).

##### Remarks.

The external male genitalia of this species are similar to those of Zeugophora (Pedrillia) annulata and Z. (P.) bicolor in that the basal 1/3 of the median lobe is tubular, but differs in having the apex of the median lobe broader and slightly curved in lateral view, the median struts > 3 per median lobe (apex of median lobe narrower, median lobe curved in lateral view, and the median struts < 3 per median lobe in Z. (P.) annulata and Z. (P.) bicolor, Figs 25–26, 30–31, respectively).

**Figures 33–40.**
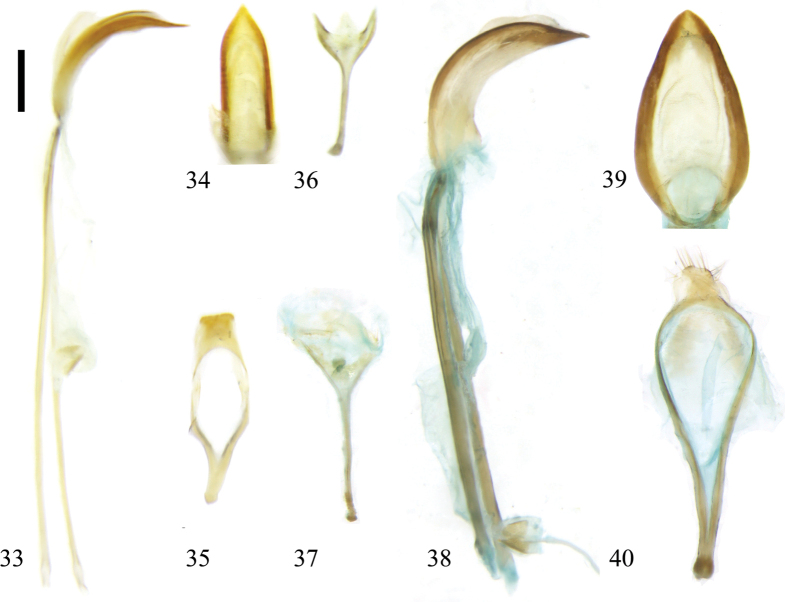
Male genitalia of Zeugophora (Pedrillia) species. **33–36.**Z. (P.) dimorpha Gressitt, 1945; **33.** Median lobe and median struts, lateral view; **34.** Median lobe, dorsal view; **35.** Tegmen, dorsal view; **36.** Spiculum, dorsal view; **37–40.**Z. (P.) emeica Li & Liang, 2018; **37.** Spiculum, dorsal view; **38.** Median lobe and median struts, lateral view; **39.** Median lobe, dorsal view; **40.** Tegmen, dorsal view. Scale bar: 0.2 mm.

**Figures 41–48.**
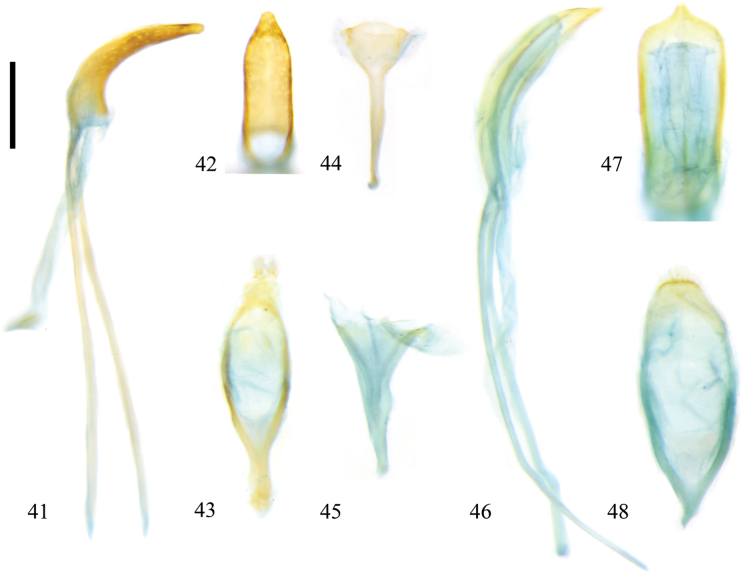
Male genitalia of Zeugophora (Pedrillia) species. **41–44.**Z. (P.) euonymorum Li & Liang, 2020; **41.** Median lobe and median struts, lateral view; **42.** Median lobe, dorsal view; **43.** Tegmen, dorsal view; **44.** Spiculum, dorsal view; **45–48.**Z. (P.) flavithorax Li & Liang, 2020; **45.** Spiculum, dorsal view; **46.** Median lobe and median struts, lateral view; **47.** Median lobe, dorsal view; **48.** Tegmen, dorsal view. Scale bar: 0.2 mm.

#### ﻿ Zeugophora (Pedrillia) longicornis

Taxon classificationAnimaliaColeopteraMegalopodidae

(Westwood, 1864)

9E54889C-B292-5A91-AD13-4F12419B9E17

Figs 53–56

Pedrillia longicornis Westwood, 1864: 280. TL: India (Bombay). TD: NHMUK.
Zeugophora andrewesi Jacoby, 1903: 82. Synonymized by Bryant 1943: 246.
Zeugophora longicornis : Bryant 1943: 246.
Zeugophora (Pedrillia) longicornis : Kimoto & Gressitt 1979: 205.

##### Host plant.

Lamiaceae: *Vitex quinata* (Lour.) Will. (pers. obs.).

##### Remarks.

The external male genitalia of this species differ from all other species of Zeugophora (Pedrillia) in having the median lobe short and broad, strongly curved in lateral view, apical portion strongly upward curved, apex sharp (Figs 54–55).

**Figures 49–56.**
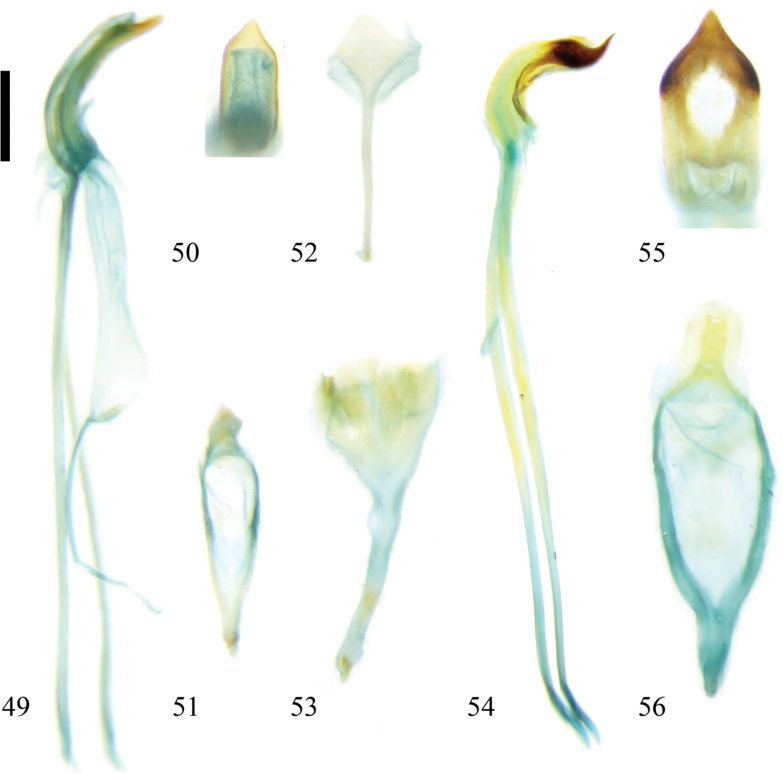
Male genitalia of Zeugophora (Pedrillia) species. **49–52.**Z. (P.) indica Jacoby, 1903; **49.** Median lobe and median struts, lateral view; **50.** Median lobe, dorsal view; **51.** Tegmen, dorsal view; **52.** Spiculum, dorsal view; **53–56.**Z. (P.) longicornis (Westwood, 1864); **53.** Spiculum, dorsal view; **54.** Median lobe and median struts, lateral view; **55.** Median lobe, dorsal view; **56.** Tegmen, dorsal view. Scale bar: 0.2 mm.

#### ﻿ Zeugophora (Pedrillia) maculata

Taxon classificationAnimaliaColeopteraMegalopodidae

Chûjô, 1941

CFB80E34-4122-5A6D-85C0-40F9EE842FDB

Figs 57–59

Zeugophora maculata , Chûjô, 1941: 463, 499. TL: China (Taiwan). TD: unknown.

##### Host plant.

Symplocaceae: *Symplocos sumuntia* Buch.-Ham. ex D. Don (pers. obs.).

##### Remarks.

**Figures 57–63.**
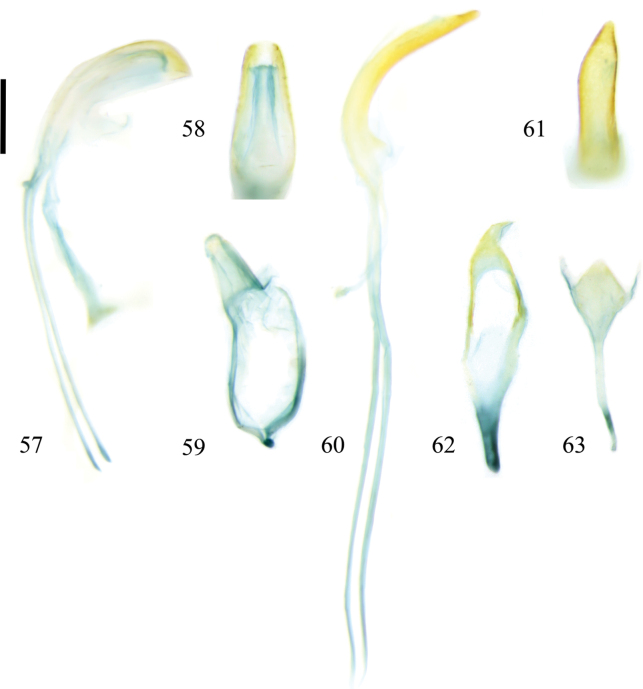
Male genitalia of Zeugophora (Pedrillia) species. **57–59.**Z. (P.) maculata Chûjô, 1941; **57.** Median lobe and median struts, lateral view; **58.** Median lobe, dorsal view; **59.** Tegmen, dorsal view; **60–63.**Z. (P.) nigricollis (Jacoby, 1885); **60.** Median lobe and median struts, lateral view; **61.** Median lobe, dorsal view; **62.** Tegmen, dorsal view; **63.** Spiculum, dorsal view. Scale bar: 0.2 mm.

The external male genitalia of this species differ from other species of Zeugophora (Pedrillia) in having the apical margin of median lobe blunt and medially downward (Figs 57–58).

#### ﻿ Zeugophora (Pedrillia) nigricollis

Taxon classificationAnimaliaColeopteraMegalopodidae

(Jacoby, 1885)

A5EB7265-8AB5-5F2F-972C-4C44CD600B2F

Figs 60–63

Pedrillia nigricollis Jacoby, 1885: 195. TL: Japan, Wada-tôge. TD: NHMUK. Synonymized as Pedrillia bicolor Kraatz, 1879a: 120 by Kimoto 1986: 309.
Zeugophora (Pedrillia) nigricollis : Crowson 1946: 95; Gressitt & Kimoto 1961: 24, 27; Chûjô 1954: 51; Chûjô & Kimoto 1961: 119; Kimoto 1964: 108.
Zeugophora (Pedrillia) bicolor : Kimoto 1986: 309; Jolivet 1957: 12; Kimoto & Takizawa 1994: 6, 99, 267; An & Kwon 2002: 272; Silfverberg 2010: 334; Li & Liang 2018: 133.
Zeugophora bicolor : Gressitt 1945: 139; An & Kwon 1995: 91–92; Takizawa 2006: 2; Rodríguez-Mirón 2018: 291.
Zeugophora nigricollis (Jacoby, 1885). Restored as a valid species by Takemoto 2019: 15–19.

##### Host plants.

Celastraceae: *Euonymus sieboldiana* Blum., *Euonymus alatus* (Thunb.) Siebold (Takemoto 2019; Li and Liang 2020).

##### Remarks.

The external male genitalia of this species are similar to those of Zeugophora (Pedrillia) annulata, Z. (P.) bicolor, Z. (P.) tricolor, and Z. (P.) euonymorum, but differ from Z. (P.) annulata, Z. (P.) bicolor, and Z. (P.) tricolor in having the median lobe less curved in the lateral view and slender, with the apex of the median lobe triangular and blunt; genitalia differ from that of Z. (P.) euonymorum in having the median lobe slender and the apex slightly narrower.

#### ﻿ Zeugophora (Pedrillia) nigroapica

Taxon classificationAnimaliaColeopteraMegalopodidae

Li & Liang, 2018

94AFA0CF-D043-52C8-A2F8-D74B4D099D04

Figs 64–67

Zeugophora (Pedrillia) nigroapica Li & Liang, 2018: 143. TL: China (Yunnan). TD: IZCAS.

##### Host plant.

Unknown.

##### Remarks.

The similarities and differences of external male genitalia between this species and Zeugophora (Pedrillia) emeica Li & Liang are presented in the remarks of Z. (P.) emeica. The external male genitalia of this species differ from other species of the subgenus Pedrillia except that of Z. (P.) emeica in having a sub-oval median lobe.

#### ﻿ Zeugophora (Pedrillia) tricolor

Taxon classificationAnimaliaColeopteraMegalopodidae

Chen & Pu, 1962

A4B7FF63-9E73-5EDD-83EC-6DC79F27CDF2

Figs 68–71

Zeugophora (Pedrillia) tricolor Chen & Pu, 1962: 116, fig. 2. TL: China (Gansu). TD: IZCAS.

##### Host plant.

Celastraceae: *Euonymus alatus* (Thunb.) Siebold (pers. obs.).

##### Remarks.

The similarities and differences of external male genitalia of this species, Zeugophora (Pedrillia) annulata, and Z. (P.) bicolor are described in the remarks under Z. (P.) annulata and Z. (P.) bicolor.

#### ﻿ Zeugophora (Pedrillia) trifasciata

Taxon classificationAnimaliaColeopteraMegalopodidae

Li & Liang, 2020

4380BAAE-8503-5BB6-90CA-B9DC09F86D90

Figs 72–75

Zeugophora (Pedrillia) trifasciata Li & Liang, 2020, ZooKeys, 975: 69–72, figs 58–66. TL: China: Yunnan. TD: KIZ.

##### Host plant.

Unknown.

##### Remarks.

The similarities and differences of external male genitalia between this species and Zeugophora (Pedrillia) dimorpha are described in the remarks under Z. (P.) dimorpha.

The external male genitalia of this species are similar to that of Zeugophora (Pedrillia) yunnanica Chen & Pu in shape, but differ in having the median lobe dorso-central with a strongly sclerotized sclerite (without a strongly sclerotized sclerite in Z. (P.) yunnanica, Fig. 82).

**Figure 64–71.**
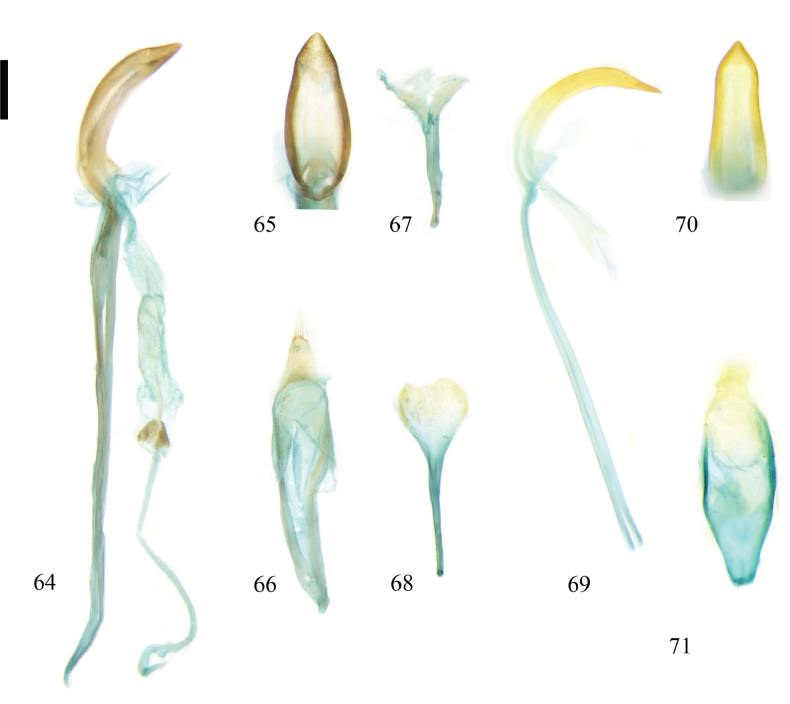
Male genitalia of Zeugophora (Pedrillia) species. **64–67.**Z. (P.) nigroapica Li & Liang, 2018; **64.** Aedeagus, lateral view; **65.** Median lobe, dorsal view; **66.** Tegmen, dorsal view; **67.** Spiculum, dorsal view; **68–71.**Z. (P.) tricolor Chen & Pu, 1962; **68.** Spiculum, dorsal view; **69.** Median lobe and median struts, lateral view; **70.** Median lobe, dorsal view; **71.** Tegmen, dorsal view. Scale bar: 0.2 mm.

#### ﻿ Zeugophora (Pedrillia) yuae

Taxon classificationAnimaliaColeopteraMegalopodidae

Li & Liang, 2020

248E1D73-DA8E-56EA-A4D1-6353B9584842

Figs 76–79

Zeugophora (Pedrillia) yuae Li & Liang, 2020, ZooKeys, 975: 72–76, figs 67–79. TL China: Yunnan. TD: KIZ, IZCAS.

##### Host plant.

Lamiaceae: *Vitex quinata* (Lour.) Will. (Li and Liang 2020).

##### Remarks.

The external male genitalia of this species are similar to that of Zeugophora (Pedrillia) tricolor in shape, but differ in having the median lobe less curved in the lateral view, the apex of the median lobe slightly broader and blunt, and the ratio of median struts / median lobe ~3.5 (the median lobe is more curved in the lateral view, the apex of the median lobe slightly narrower and sharper, and the ratio of median struts / median lobe is ~2.2 in Z. (P.) tricolor).

**Figures 72–75.**
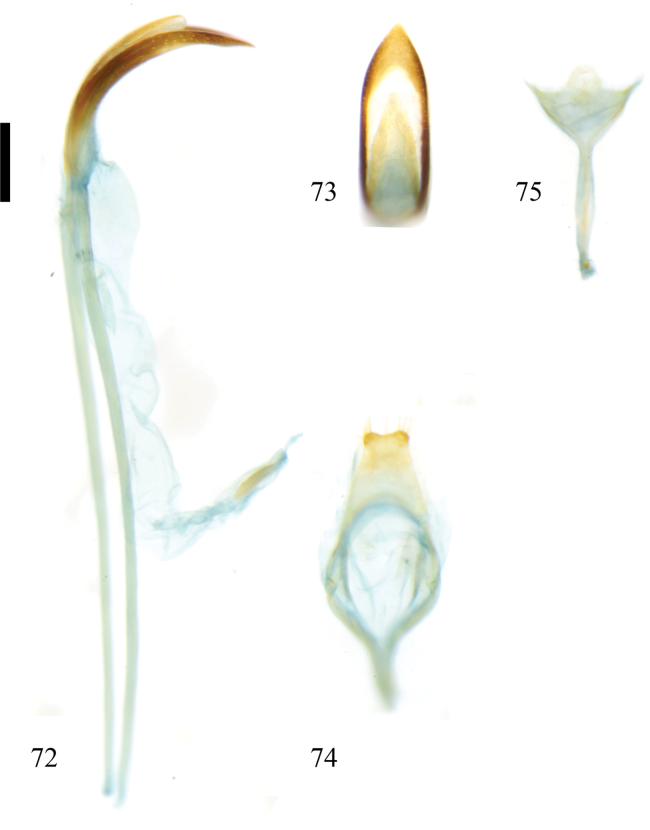
Male genitalia of Zeugophora (Pedrillia) trifasciata Li & Liang, 2020. **72.** Median lobe and median struts, lateral view; **73.** Median lobe, dorsal view; **74.** Tegmen, dorsal; **75.** Spiculum, dorsal view. Scale bar: 0.2 mm.

#### ﻿ Zeugophora (Pedrillia) yunnanica

Taxon classificationAnimaliaColeopteraMegalopodidae

Chen & Pu, 1962

5A17C5E3-F81F-5B67-8422-60CD34C4B5A0

Figs 80–83

Zeugophora (Pedrillia) yunnanica Chen & Pu, 1962: 116, fig. 3. TL: China (Yunnan). TD: IZCAS.

##### Host plant.

Symplocaceae: *Symplocos* sp. (Chen and Pu 1962).

##### Remarks.

The similarities and differences of external male genitalia of this species, Zeugophora (Pedrillia) dimorpha, and Z. (P.) trifasciata are presented in the remarks under Z. (P.) dimorpha and Z. (P.) trifasciata.

**Figures 76–83.**
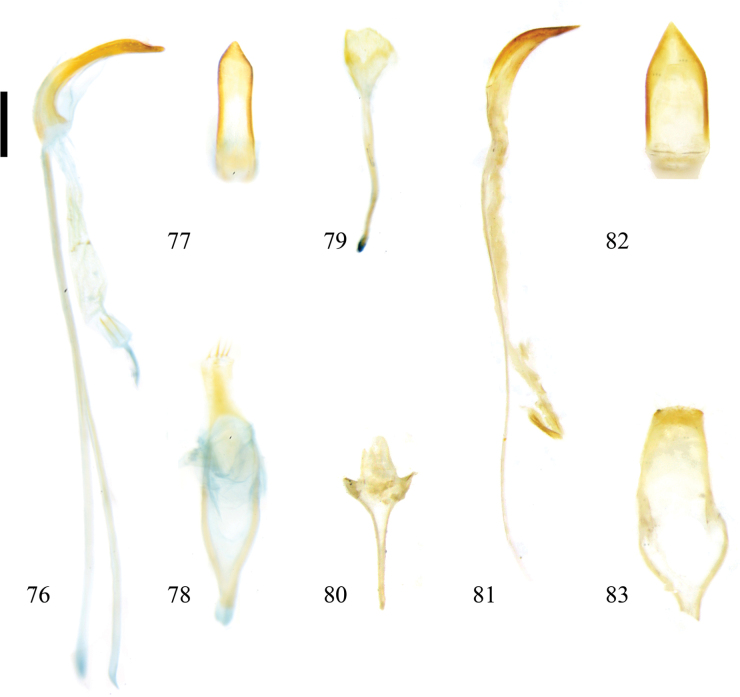
Male genitalia of Zeugophora (Pedrillia) species. **76–79.**Z. (P.) yuae Li & Liang, 2020; **76.** Median lobe and median struts, lateral view; **77.** Median lobe, dorsal view; **78.** Tegmen, dorsal view; **79.** Spiculum, dorsal view; **80–83.**Z. (P.) yunnanica Chen & Pu, 1962; **80.** Spiculum, dorsal view; **81.** Median lobe and median struts, lateral view; **82.** Median lobe, dorsal view; **83.** Tegmen, dorsal view. Scale bar: 0.2 mm.

#### ﻿ Zeugophora (Pedrillia) liupanshanensis sp. nov.

Taxon classificationAnimaliaColeopteraMegalopodidae

2642BC73-04A7-5D3D-B0DF-9A6906DDC966

https://zoobank.org/1751E637-1A2B-4A46-8740-B2AC4DCD64E8

Suggested Chinese name 六盘山小距甲 Figs 84–91, 92–99, 100–102, 103–106

##### Specimens examined.

***Holotype*** (KIZ): male, China, Ningxia, Jingyuan, Liupan Shan National Nature Reserve, Danangou, 35.48659°N, 106.27045°E / 2130 m, 2018.vii.28, Kaiqin Li coll., Kunming Institute of Zoology, Chinese Acad. Sci. / Holotype, Zeugophora (Pedrillia) liupanshanensis sp. nov., des. by K.Q. Li & H.B. Liang, 2024 [red label]. ***Paratype*** (IZCAS): 1 male, China, Ningxia, Jingyuan, Liupan Shan National Nature Reserve, Longtan Forest Farm, 35.39160°N, 106.35299°E / 1918 m, 2018.vii.26, Kaiqin Li & Likun Zhang coll., Kunming Institute of Zoology, Chinese Acad. Sci. / Paratype, Zeugophora (Pedrillia) liupanshanensis sp. nov., des. by K.Q. Li & H.B. Liang, 2024 [yellow label].

**Figures 84–91.**
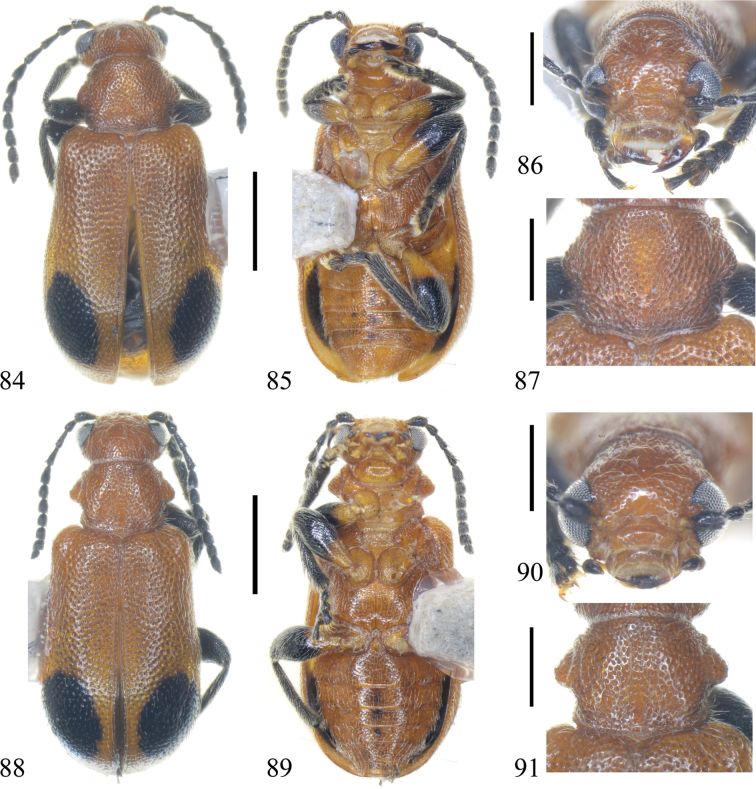
Holotype and paratype of Zeugophora (Pedrillia) liupanshanensis sp. nov. **84–87.** Holotype, male; **84.** Dorsal view; **85.** Ventral view; **86.** Head, anterior view; **87.** Pronotum, dorsal view; **88–91.** Paratype, male; **88.** Dorsal view; **89.** Ventral view; **90.** Head, anterior view; **91.** Pronotum, dorsal view. Scale bars: 1.0 mm (**84, 85, 88, 89**); 0.5 mm (**86, 87, 90, 91**).

##### Diagnosis.

Head brown, pronotum, scutellum, and underside brown; elytra brown, posterior portion with two black spots; antennae black; legs brown with posterior portion black; antennomeres 3 and 4 long, 5–11 short; apex of median lobe triangular and narrow.

##### Description.

BL = 3.8 mm, BW = 1.7 mm. Head brown, except posterior portion of mandible black; pronotum, scutellum, and underside brown; elytra brown, but with two black spots posteriorly; antennae black; legs brown but distal femora, tibiae, and tarsi black.

**Head**: eye prominent, inner margin with distinct canthus; vertex sparsely punctate and pubescent centrally, densely punctate and pubescent laterally; occiput strongly constricted; frons sparsely punctate and pubescent, center with a shallow concave; clypeus rectangular, width 2× length, punctate and pubescent laterally and anteriorly, separated from frons by deep clypeal suture; labrum rectangular, narrower than clypeus, anterior margin with punctures and pubescence; antennae slender, exceeding humeri in length, antennomere 1 long and swollen, antennomere 2 short, 2/3 as long as antennomere 1, antennomere 3 as long as 4 and 1, antennomere 5 shorter than antennomere 4, antennomeres 6–10 as long as antennomere 2, antennomere 11 acute at apex, as long as antennomere 5; antennomeres 1–4 sparsely punctate and pubescent, antennomeres 5–11 densely punctate and pubescent.

**Figures 92–99.**
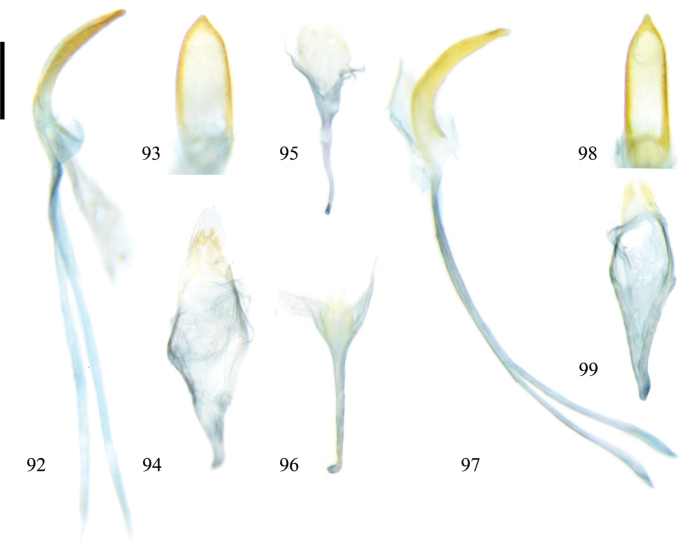
Holotype and paratype of Zeugophora (Pedrillia) liupanshanensis sp. nov. **92–95.** Male genitalia of holotype; **92.** Median lobe and median struts, lateral view; **93.** Median lobe, dorsal view; **94.** Tegmen, dorsal view; **95.** Spiculum, dorsal view; **96–99.** Male genitalia of paratype; **96.** Spiculum, dorsal view; **97.** Median lobe and median struts, lateral view; **98.** Median lobe, dorsal view; **99.** Tegmen, dorsal view. Scale bar: 0.2 mm.

**Thorax**: PW/PL = 1.3; anterior margin slightly flattened; posterior margin extended posteromedially ; length of posterior margin slightly longer than anterior margin; anterior groove distinct laterally, obsolete medially; posterior groove deep laterally, shallow medially; anterior portion of lateral margin subparallel, then gradually expanding from anterior portion to middle, strongly constricted behind middle; lateral tubercle rounded; behind the lateral tubercle, an oblique groove extending to basal portion; disc convex with dense and coarse punctures and pubescence, basal portion slightly depressed; basal portion of each side slightly prominent. Scutellum triangular, slightly emarginate at apex, densely punctate and pubescent.

**Figures 100–102.**
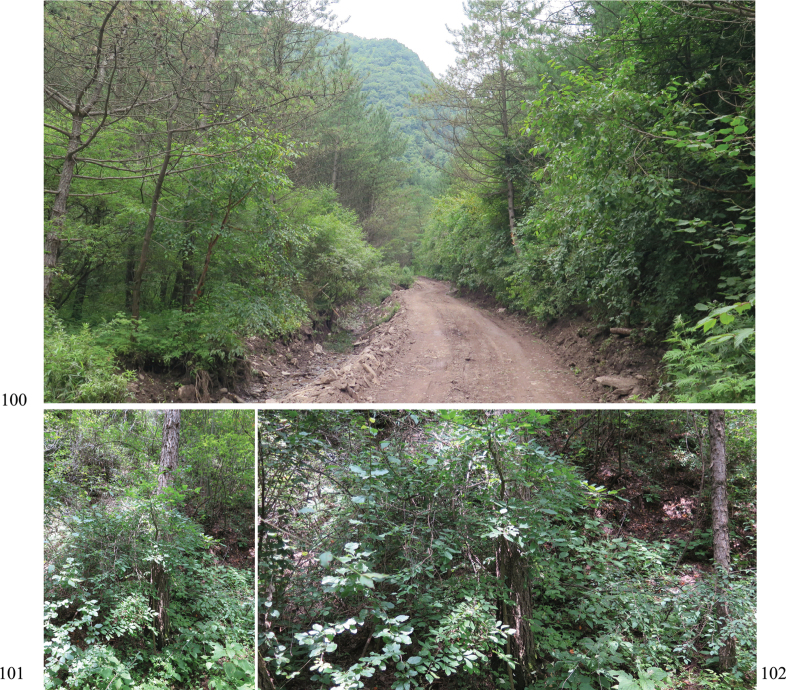
Habitat of Zeugophora (Pedrillia) liupanshanensis sp. nov. (Ningxia, Liupan Shan, Danangou). **100.** Habitat; **101, 102.** Host plants *Euonymus przewalskii* (Celastraceae) of Z. (P.) liupanshanensis sp. nov.

**Elytra**: EL/EW = 1.5–1.6; elytral humeri projecting antero-laterally, humeral groove shallow, lateral part of humeri densely punctate and pubescent; lateral margin slightly expanding from the base to the middle, widest behind the middle, apex slightly rounded; disc slightly flattened, coarsely punctate and pubescent; suture with one row of punctures and pubescence; epipleura narrow, two rows of punctures and pubescence at base and one row at apex.

**Abdomen and legs**: underside sparsely punctate and pubescent. Legs moderately long, femora robust, mid- and hind-tibiae slightly curved. Pygidium moderately long, apical portion exposed. Apical margin of last abdominal ventrite slightly prominent. Median lobe sclerotized, short and broad, curved in lateral view, dorso-central portion membranous, basal 1/3 tubular, sides thickened and parallel, apex triangular, strongly constricted and slightly sharp; median struts rod-shaped, widely separated from each other, ~2.2–2.4× as long as median lobe; basal portion of tegmen Y-shaped, tegminal ring subdiamond-shaped, paramere tongue-shaped, apical margin of paramere with setae; endophallus membranous, with weakly sclerotized area. Spiculum spoon-shaped, apical margin prominent.

##### Distribution.

China (Ningxia).

##### Host plants.

*Euonymus przewalskii*, *E. giraldii* (Celastraceae). We collected this species by beating the Celastraceae trees in the forest. One specimen was collected from *E. giraldii*, the other was from *E. przewalskii*. Several leaves were observed to have damage from bite marks (Fig. 106).

##### Etymology.

The specific name *liupanshanensis* refers to the type locality, Liupan Shan.

##### Remarks.

The pronotum shape of the new species is similar to that of Zeugophora (Pedrillia) eumonymorum Li & Liang, 2020 which is also found in Liupan Shan, but differs from the latter in having body mostly brown elytra with black spots, shorter antennae, the apex of the median lobe narrower (vs body dark blue or green, underside black, longer antennae, apex of median broader in Z. (P.) eumonymorum).

The external male genitalia of this species are similar to those of Zeugophora (Pedrillia) annulata, Z. (P.) bicolor, and Z. (P.) tricolor (Figs 25, 26, 30, 31, 69, 70), but differ from them in having a less curved median lobe in lateral view, and a shorter and less sclerotized apex of the median lobe, (more curved median lobe in lateral view, apex of median lobe longer and more sclerotized in Z. (P.) annulata, Z. (P.) bicolor, and Z. (P.) tricolor).

**Figures 103–106.**
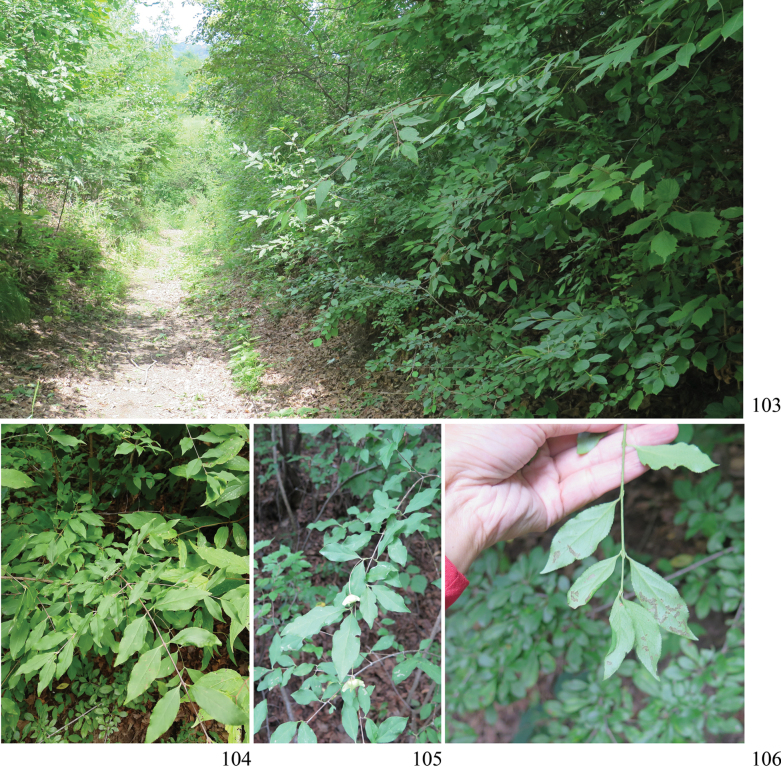
Habitat and host plants of Zeugophora (Pedrillia) liupanshanensis sp. nov. (Ningxia, Liupan Shan, Longtan forest farm). **103.** Habitat; **104–106.** Host plant *Euonymus giraldii* (Celastraceae) of Z. (P.) liupanshanensis sp. nov.; **106.** Feeding traces on the abaxial leaf surface caused by adults.

This species is similar to Zeugophora (Zeugophora) tetraspilota Medvedev, 1998 (Fig. 110) in having posterior spots on elytra, but differs in having brown elytra with two black spots, a brown pronotum, black antennae, brown legs with black coloration, and a broader lateral tubercle on the pronotum (elytra yellowish brown with four black spots; pronotum, antennae, and legs yellowish brown, narrower lateral tubercle on the pronotum in *Z. tetraspilota*).

This species is also similar to Zeugophora (Pedrillia) trisignata An & Kwon, 2002 and Z. (P.) bimaculata Kratz, 1879 in having posterior spots on the elytra and in body shape, but differs from the latter in having a brown head, black antennae, brown legs with the posterior portion black, and antennomeres 3 and 4 longer (vs black head, antennomeres 1–4 yellowish brown, yellowish brown legs, antennomeres 3–4 shorter in *Z. trisignata* and *Z. bimaculata*, Figs 108, 109, respectively). The external male genitalia of this species differ from that of Z. (P.) bimaculata in having a narrower apex of the median lobe (apex of median lobe broader in Z. (P.) bimaculata).

Moreover, we found the elytra colorations of Z. (P.) bimaculata and Z. (P.) trisignata to be variable. The species Z. (P.) bimaculata has three kinds of coloration (Sergeev and Legalov 2022): one of them is the same as the type specimen of Z. (P.) trisignata (type locality Odaesan, Korea). The species Z. (P.) trisignata has two kinds of coloration (An and Kim 2020), of which one is the same as the type of Z. (P.) bimaculata (type locality Amur, Russia). Therefore, we compared descriptions and illustrations of the types of Z. (P.) bimaculata (Kraatz, 1879b; Fig. 108) and Z. (P.) trisignata (An and Kwon 2002; Fig. 109) as no specimens were available, and no significant difference was found in external morphology. We speculate Z. (P.) trisignata is probably conspecific with Z. (P.) bimaculata, but their genitalia should be compared to confirm this synonymy.

**Figures 107–110.**
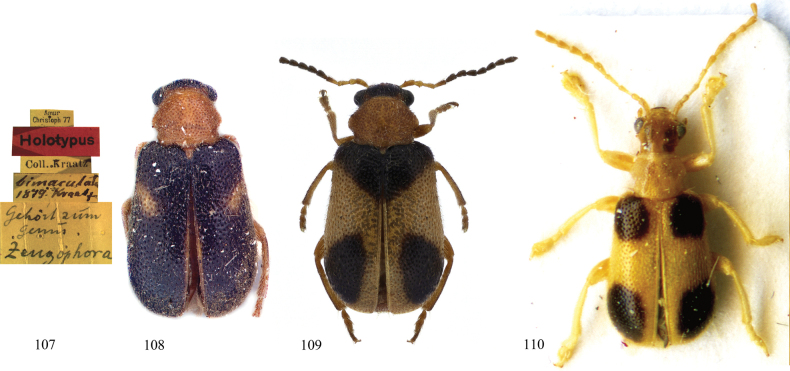
Types of *Zeugophora*. **107, 108.** Type of Z. (Pedrillia) bimaculata Kraatz, 1879; **109.** Holotype of Z. (Pedrillia) trisignata An & Kwon, 2002; **110.** Type of Z. (Zeugophora) tetraspilota Medvedev, 1998.

## ﻿Discussion

In recent years, we collected many specimens of Chinese Zeugophorinae during expeditions, also examining their biology and host plants. We observed that species having host plants of the same genus or family have similar external male genitalia. For example, in the subgenus Zeugophora, the host plants all belong to the family Salicaceae (Chen 1974; Wu et al. 1982; Bukejs 2009; Sergeev and Legalov 2022); the species studied in this article have the similar characteristics of external male genitalia, such as the ratio of median strut / median lobe ≤ 1.5, the trifid apex of spiculum. The host plants of *Zeugophora hozumii* Chûjô, 1953, *Z. japonica* Chûjô, 1951, and *Z. cupka* Takemoto, 2019 from Japan and Russia are also species of the family Salicaceae (Takemoto 2019), and their external male genitalia have similar characteristics, the ratio of median strut / median lobe ≤ 1.5 and the trifid apex of spiculum.

In the subgenus Pedrillia, the host plants of Zeugophora (Pedrillia) annulata, Z. (P.) bicolor, Z. (P.) tricolor, Z. (P.) nigricollis, Z. (P.) euonymorum, and Z. (P.) liupanshanensis sp. nov. are Celastraceae, and those species have external male genitalia similar to each other and differing from species of *Zeugophora*, such as a membranous dorso-central portion of the median lobe with its tubular basal third, parallel sides, a tongue-shaped apical portion of the tegmen, and a prominent apex of the spiculum that is not trifid. According to Takemoto (2019), *Zeugophora unifasciata* (Jacoby, 1885), *Z. chujoi* Ohno, 1961, and *Z. flavonotata* (Chûjô, 1935) share similarities in their median lobe, tegmen, and spiculum with the Chinese species that feed on Celastraceae, and all of them are also recorded to have Celastraceae as their host plants.

The species Zeugophora (Pedrillia) yunnanica, Z. (P.) trifasciata, Z. (P.) dimorpha, and Z. (P.) maculata, Z. (P.) flavithorax have similar external male genitalia, such as the membranous median lobe dorso-central portion without a tubular basal third, thin parallel sides, and the apex of the spiculum usually trifid. Now that we know that the host plants of Z. (P.) yunnanica, Z. (P.) maculata, and Z. (P.) flavithorax are Symplocaceae, it explains the similarities. Other species such as *Zeugophora gede* Reid, 1998 from Indonesia and *Zeugophora varipes* Jacoby, 1885 from Japan also have similar external male genitalia to the three Chinese species, and their host plants are also Symplocaceae (Reid 1998, Takemoto 2019). Based on the similarity in genitalia, we speculate the host plant of Z. (P.) dimorpha and Z. (P.) trifasciata is probably also Symplocaceae.

Based on our observations, characteristics of external morphs, male genitalia, and information on host plants need to be examined together, to provide a more accurate classification of the Zeugophorinae.

## Supplementary Material

XML Treatment for Subgenus Zeugophora

XML Treatment for Zeugophora (Zeugophora) ancora

XML Treatment for Zeugophora (Zeugophora) cribrata

XML Treatment for Zeugophora (Zeugophora) cyanea

XML Treatment for Zeugophora (Zeugophora) nigroaerea

XML Treatment for Zeugophora (Zeugophora) scutellaris

XML Treatment for Zeugophora (Zeugophora) turneri

XML Treatment for Subgenus Pedrillia

XML Treatment for Zeugophora (Pedrillia) annulata

XML Treatment for Zeugophora (Pedrillia) bicolor

XML Treatment for Zeugophora (Pedrillia) dimorpha

XML Treatment for Zeugophora (Pedrillia) emeica

XML Treatment for Zeugophora (Pedrillia) euonymorum

XML Treatment for Zeugophora (Pedrillia) flavithorax

XML Treatment for Zeugophora (Pedrillia) indica

XML Treatment for Zeugophora (Pedrillia) longicornis

XML Treatment for Zeugophora (Pedrillia) maculata

XML Treatment for Zeugophora (Pedrillia) nigricollis

XML Treatment for Zeugophora (Pedrillia) nigroapica

XML Treatment for Zeugophora (Pedrillia) tricolor

XML Treatment for Zeugophora (Pedrillia) trifasciata

XML Treatment for Zeugophora (Pedrillia) yuae

XML Treatment for Zeugophora (Pedrillia) yunnanica

XML Treatment for Zeugophora (Pedrillia) liupanshanensis

## ﻿Additional information

### Use of AI

No use of AI was reported.

### Author ORCIDs

Kai-Qin Li https://orcid.org/0000-0001-7854-8445

Yuan Xu https://orcid.org/0009-0000-3103-9125

Hong-Bin Liang https://orcid.org/0000-0002-9668-1167

### Data availability

All of the data that support the findings of this study are available in the main text.

## References

[B1] An SL Kim E (2020) A guide book of Korean Leaf Beetles. Nature & Ecology, Paju, 400 pp. [In Korean]

[B2] An SL Kwon YJ (2002) Classification of the Leaf beetles from Korea Part IV. Subfamily Zeugophorinae (Coleoptera: Chrysomelidae). Insecta Koreana 19(3, 4): 271–276.

[B3] Baly JS (1873) Catalogue of the phytophagous Coleoptera of Japan, with descriptions of the species new to science. Transactions of the Royal Entomological Society of London 1873(1): 69–99. 10.1111/j.1365-2311.1873.tb00637.x

[B4] Bryant E (1943) New species of African *Zeugophora* (Orsodacninae, Col.) and synonymy. Annals & Magazine of Natural History 10(64): 245–248. 10.1080/03745481.1943.9728014

[B5] Bukejs A (2009) Data on species of Megalopodidae and Orsodacnidae (Coleoptera: Chrysomeloidea) in Latvian fauna. Acta Biologica Universitatis Daugavpiliensis 9(1): 73–78.

[B6] Chen SH (1940) Attempt at a new classification of the leaf beetles. Sinensia 11(5–6): 451–481.

[B7] Chen SH (1974) New chrysomelid beetles from West China. Acta Entomologica Sinica 17(1): 43–48.

[B8] Chen SH Pu FJ (1962) Notes on Chinese Megalopodinae. Acta Entomologica Sinica 11(Suppl.): 114–118.

[B9] Chûjô M (1935) Chrysomelidae of Loo-Choo Archipelago (I). Transactions of the Natural History Society of Formosa Taihoku 25: 136–139.

[B10] Chûjô M (1937) Descriptions of Four New Species of the Genus *Pedrillia* Westwood, with a list of the species known from the World (Col. Chrysomelidae). Transactions of the Natural History Society of Formosa Taihoku 27: 146–151.

[B11] Chûjô M (1941) Description of new chrysomelid beetles from Formosa (I). Transactions of the Natural History Society of Formosa 31: 463–467.

[B12] Chûjô MA (1952) Taxonomic study on the Chrysomelidae (Insecta-Coleoptera) from Formosa. Part IV Subfamily Zeugophorinae. The Technical Bulletin of the Kagawa Agricultural College 3(3): 166–183.

[B13] Chûjô MA (1953) A taxonomic study on the Chrysomelidae with special reference to the fauna of Formosa. The Technical Bulletin of the Kagawa Agricultural College 5(2): 121–136.

[B14] Chûjô M (1954) Chrysomelid-beetles from Shikoku, Japan (III). Transactions of the Shikoku Entomological Society 4: 51–62.

[B15] Chûjô M (1959) Contribution to the fauna of Chrysomelidae (Coleoptera) in Japan (III). Memoirs of the Faculty of Liberal Arts & Education. Kagawa University 81(part II): 1–16.

[B16] Chûjô M Kimoto S (1961) Systematic catalog of Japanese Chrysomelidae (Coleoptera). Pacific Insects 3: 117–202.

[B17] Crowson RA (1946) A revision of the genera of the Chrysomelid group Sagrinae (Coleoptera). Transactions of the Royal Entomological Society of London 97(4): 75–115. 10.1111/j.1365-2311.1946.tb00276.x

[B18] Gressitt JL (1945) On some genera of Oriental Orsodacninae and Eumolpinae (Col. Chrysom.). Lingnan Science Journal 21: 135–146.

[B19] Gressitt JL Kimoto S (1961) The Chrysomelidae (Coleopt.) of China and Korea, Part 1. Pacific Insects Monograph 1A: 1–299.

[B20] Jacoby M (1885) Descriptions of the phytophagous Coleoptera of Japan, obtained by Mr. George Lewis during his second journey, from February 1880 to September 1881. Part I. Proceedings of the Zoological Society of London 1885(1): 190–211 [pl. XI]. 10.1111/j.1096-3642.1885.tb02894.x

[B21] Jacoby M (1903) Descriptions of the new genera and species of Phytophagous Coleoptera obtained by Mr H. L. Andrewes and Mr T. R. D. Bell at the Nilgiri hills and Kanara. Annales de la Société Entomologique de Belgique 47: 80–128.

[B22] Jacoby M (1908) The Fauna of British India, including Ceylon and Burma. Coleoptera, Chrysomelidae. Vol. 1. Taylor & Francis, London, [XX +] 534 pp. [2 pls]

[B23] Jolivet P (1957) Chrysomelidae: Orsodacninae. In: Hincks WD (Ed.) Coleopterorum Catalogus Supplementa, Pars 51, Fasc. 3. W. Junk, s-Gravenhage, 16 pp.

[B24] Kasap H Crowson A (1985) The studies on the ovipositors and 8^th^ abdominal segments of species of Bruchidae and Chrysomelidae (Coleoptera). Türkiye Bitkiler kor. Dergisi 9(3): 131–145.

[B25] Kimoto S (1964) The Chrysomelidae of Japan and the Ryukyu Islands. I & VI. Journal of the Faculty of Agriculture Kyushu University Fukuoka 13: 99–164, 235–308. 10.5109/22722

[B26] Kimoto S (1986) New or little known Chrysomelidae (Coleoptera) from Japan and its adjacent regions, 4. Entomological papers presented to Yoshihiko Kurosawa on the occasion of his retirement. Coleopterists’ Association of Japan, Tokyo, 309–313.

[B27] Kimoto S Gressitt J (1979) Chrysomelidae (Coleoptera) of Thailand, Cambodia, Laos, and Vietnam. 1. Sagrinae, Donaciinae, Zeugphorinae, Megalopodinae and Criocerinae. Pacific Insects 20(2–3): 191–256.

[B28] Kimoto S Takizawa H (1994) Leaf Beetles (Chrysomelidae) of Japan. Tokai University Press, Tokyo, [XVII +] 539 pp. [133 pls]

[B29] Kraatz G (1871) Eine neue deutsche Criocerinen-Art. Berliner Entomologische Zeitschrift 15(2–3): 162. 10.1002/mmnd.18710150212

[B30] Kraatz G (1879a) Neue ostsbirische Arten der Chrysomelinen-Gattung *Pedrillia*. Deutsche Entomologische Zeitschrift 23: 119–120. 10.1002/mmnd.48018790116

[B31] Kraatz G (1879b) Neue Käfer vom Amur. Deutsche Entomologische Zeitschrift 23: 121–144 [pl. II]. 10.1002/mmnd.48018790117

[B32] Kuschel G May BM (1990) Palophaginae, a new subfamily for leaf-beetles, feeding as adult and larva on Araucarian pollen in Australia (Coleoptera: Megalopodidae). Invertebrate Taxonomy 3(6): 697–719. 10.1071/IT9890697

[B33] Lee CF Cheng HT (2007) The Chrysomelidae of Taiwan 1. Sishou-Hills Insect Observation Network Press, Taipei, 199 pp. [In Chinese]

[B34] Li KQ Liang HB (2018) A check list of the Chinese Zeugophorinae (Coleoptera: Megalopodidae), with new synonym, new record and two new species of subgenus Pedrillia from China. Zootaxa 4455(1): 127–149. 10.11646/zootaxa.4455.1.5 30314223

[B35] Li KQ Liang HB (2020) Four new species and two new records of genus *Zeugophora* (Coleoptera, Megalopodidae, Zeugophorinae) from China. ZooKeys 975: 51–78. 10.3897/zookeys.975.53472 33117064 PMC7572520

[B36] Li KQ Liang ZL Liang HB (2013) Two new species of the genus *Temnaspis* Lacordaire, 1845, (Coleoptera: Chrysomeloidea: Megalopodidae) from China and Myanmar, with notes on the biology of the genus. Zootaxa 3737(4): 379–398. 10.11646/zootaxa.3737.4.3 25112760

[B37] Lopatin IK (2008) New species of the leaf beetles (Coleoptera, Chrysomelidae) from China. IX. Entomological Review 88(8): 918–927. 10.1134/S001387380808006X

[B38] Mann JS Crowson RA (1996) Internal Sac structure and Phylogeny of Chrysomelidae. In: Jolivet P Cox ML (Eds) Chrysomelidae Biology, Vol.1, Classification, Phylogeny and Genetics. Academic Publishing, Amsterdam, 291–316.

[B39] Medvedev LN (1985) Zeugophorinae and Megalopodinae (Coleoptera, Chrysomelidae) fauna of Vietnam. Insects of Vietnam 1985: 59–63.

[B40] Medvedev LN (1997) To the knowledge of Zeugophorinae (Chrysomelidae) of the Old World. Russian Entomological Journal 6(3–4): 65–69.

[B41] Medvedev LN Roginskaya EY (1988) Catalog of host-plants of leaf beetles of the USSR. SIEE RAS, Moscow, 190 pp.

[B42] Monrós F (1959) Notas sobre Chrysomelidae (Coleoptera). Acta Zoológica Lilloana 17: 1–24.

[B43] Pic M (1906) Habitats et descriptions de divers coléoptères paléarctiques. L’Échange, Revue Linnéenne 22: 25–27, 33–35, 41–42.

[B44] Pic M (1945) Coléoptères du globe (suite). L’Échange, Revue Linnéenne 61: 1–4, 13–16.

[B45] Power JA (1863) Description of a new British *Zeugophora*. The Zoologist 21: 8735.

[B46] Reid CAM (1989) The Australian species of the tribe Zeugophorini (Coleoptera: Chrysomelidae: Megalopodinae). General and Applied Entomology 21: 39–48.

[B47] Reid CAM (1992) A new species of *Zeugophora* Kunze from Java (Coleoptera: Chrysomelidae: Megalopodinae). Treubia 30(3): 403–408. 10.14203/treubia.v30i3.665

[B48] Reid CAM (1995) A cladistic analysis of subfamilial relationships in the Chrysomelidae senus lato (Chrysomeloidea). In: Pakaluk J Slipinski SA (Eds) Biology, phylogeny, and classification of Coleoptera papers celebrating the 80th birthday of Roy A.Crowson. Muzeum i Instytut Zoologii PAN, Warsaw, 559–631.

[B49] Reid CAM (1998) Two new species of *Zeugophora* Kunze in Indonesia (Coleoptera: Megalopodidae: Zeugophorinae). Serangga 3(1): 7–14.

[B50] Reitter E (1900) Coleopteren, gesammelt im Jahre 1898 in Chin.-Zentral-Asien von Dr. Holderer in Lahr. (Mit 1 Taf.). Wiener Entomologische Zeitung 19: 153–166. 10.5962/bhl.part.3449

[B51] Rodríguez-Mirón GM (2018) Checklist of the family Megalopodidae Latreille (Coleoptera: Chrysomeloidea) a synthesis of its diversity and distribution. Zootaxa 4434(2): 265–302. 30313186 10.11646/zootaxa.4434.2.3

[B52] Rodríguez-Mirón G Zaragoza-Caballero S Morrone JJ (2021) Phylogenetic analysis of the family Megalopodidae (Coleoptera: Chrysomeloidea): better taxon-sampling facilitates detection of new relationships and new taxa. Cladistics : The International Journal of the Willi Hennig Society 37(6): 1–40. 10.11646/zootaxa.4434.2.3 34841585

[B53] Schöller M (2009) First records of Zeugophorinae from New Caledonia with description of two new species (Coleoptera: Megalopodidae, Zeugophorinae). Entomologische Zeitschrift Stuttgart 119(5): 195–198.

[B54] Sekerka L Vives E (2013) Review of Zeugophorinae of New Guinea, with description of *Zeugophorella* gen. nov. And new synonyms of *Zeugophora* (Coleoptera: Megalopodidae). Acta Entomologica Musei Nationalis Pragae 53(2): 747–762.

[B55] Sergeev ME Legalov AA (2022) Review of leaf beetles of the family Megalopodidae (Coleoptera: Chrysomeloidea) from Siberia and the Russian Far East. Ecologica Montenegrina 57: 44–70. 10.37828/em.2022.57.6

[B56] Silfverberg H (2010) Megalopodidae: Zeugophorinae. In: Löbel I, Smetana A. (Eds) Catalogue of Palaearctic Coleoptera vol. 6 Chrysomeloidea. Apollo Books, Kirkeby Sand, Stenstrup, 334–335.

[B57] Snodgrass RE (1935) Principles of insect morphology. McGraw-Hill Book Company, New York, 667 pp.

[B58] Suffrian E (1840) Fragmente zur genauern Kenntniss deutscher Käfer. Entomologische Zeitung (Stettin) 1: 98–104.

[B59] Takemoto T (2019) Revision of the genus *Zeugophora* (Coleoptera, Megalopodidae, Zeugophorinae) in Japan. Zootaxa 4644(1): 001–062. 10.11646/zootaxa.4644.1 31717036

[B60] Verma KK (1996) Inter-subfamily relations among Chrysomelidae (Coleoptera) as suggested by organization of the male genital system. In: Jolivet P Cox ML (Eds) Chrysomelidae Biology, Vol.1, Classification, Phylogeny and Genetics. Academic Publishing, Amsterdam, 317–351.

[B61] Warchałowshi A (2010) The Palaearctic Chrysomelidae: identification keys. Volume 1. Natura optima dux Foundation, 629 pp. 10.1649/072.065.0210

[B62] Westwood JO (1864) Descriptions of some new Species of Coleopterous Insects belonging to the Eupodous Phytophaga, Natives of the Old World and Australia. Transactions of the Royal Entomological Society of London 2(3): 271–280. 10.1111/j.1365-2311.1864.tb00106.x

[B63] Wu FZ Gao ZN Guo YY (1982) Atlas of Agricultural Insect in Ningxia of China, Vol. 2. Ningxia People’s Publishing House, 265 pp. [In Chinese]

